# Microbial Partnerships of Pathogenic Oomycetes

**DOI:** 10.1371/journal.ppat.1006028

**Published:** 2017-01-26

**Authors:** Marie Larousse, Eric Galiana

**Affiliations:** Université Côte d'Azur, INRA, CNRS, ISA, Sophia Antipolis, France; The Sainsbury Laboratory, UNITED KINGDOM

## Introduction

Oomycetes are filamentous eukaryotic microorganisms among which several species are plant and animal pathogens [1,2]. Those that cause plant diseases have had great impacts on human activities such as (i) the 19th century Irish famine triggered by the potato late blight (*Phytophthora infestans*), (ii) the associated massive North American immigration [3], and (iii) the formulation of the Bordeaux mixture, which was the first fungicide to be used worldwide [4]. Because of their ability to develop resistant against chemical treatments and to bypass plant resistance genes, they still have severe economic repercussions on modern crops. To circumvent these problems, most studies of the last ten years have reported on the coevolutionary mechanisms between the plant host immune system and the oomycete effector repertoire that promotes successful infection [5,6,7,8].

**Fig 1:**
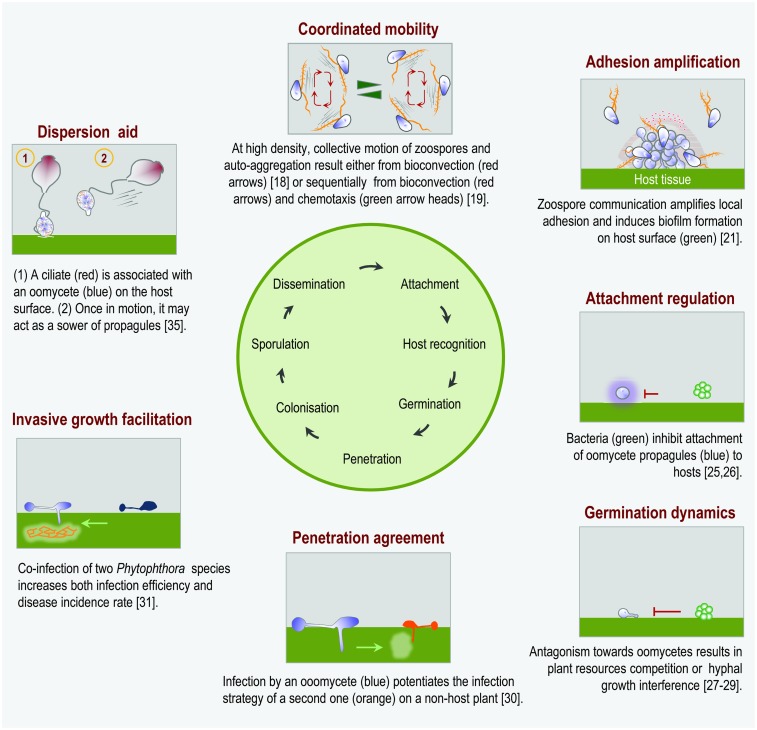
A schematic representation of phenomenological interfaces between a disease cycle and an interfering microbiota. For different steps of a disease generic cycle (central green circle), the facets of cooperation and interaction (red subtitles) are illustrated by an inset provided with a legend below it. The reference numbers of reported cases of oomycete–oomycete and microbiota–oomycete interactions cited in the text are indicated.

As for all other groups of plant pathogens, one of the current challenges is now to understand what is happening beyond the well-understood plant–oomycete interaction. To accomplish this, it is required to get a much broader picture of how the traits of the host and the pathogenic oomycete interact with the biotic environment to shape the evolution of plant resistance or oomycete pathogenicity. Concerning the host plant, the maintenance of a stable disease-resistance gene polymorphism appears to involve coevolution between the *R* gene and effector pairs but also complex and diffuse community-wide interactions [9]. The plant-associated microbiota contributes to maximize host adaptation to deal with pathogenic infection [10,11,12,13]. Concerning the pathogen, there is less understanding regarding how the pathogen–microbiota interaction accommodates the emergence of a pathogenic population, how it interferes with the expression of the effector repertoire on the plant surface, and, *in fine*, how it promotes or suppresses the disease. At the same time, an infectious entity is no longer only considered at the species level but also at the level of a resident microbiota or part thereof [14]. This paradigmatic inflexion helps (i) to unravel the molecular basis of interactions between plants and their pathogens in natural systems and (ii) to delineate the complex network of interactions that determine the spatial and temporal distribution of inocula and the genetic structure of the pathogen population as well as the communal virulence-associated mechanisms. This report highlights studies that establish how different aspects of the infectious process can be regulated by interactions between oomycetes or between oomycetes and other microbial species (Fig 1).

## How Do Zoospores Coordinate Their Motion?

The plant infection initially depends on the ability of biflagellate zoospores to reach host tissues, except for cases in which oomycete species have lost the ability to produce swimming cells. Plants emit concentration gradients of attractants, which help a zoospore in targeting optimal sites for infection [15,16]. Within the *Phytophthor*a genus, zoospores may also adopt collective motion. In Petri dishes and in the absence of chemical or electrical signals, they form plumes with cell concentrations increasing over time, a phenomenon named pattern swimming or auto-aggregation. An analysis of *P*. *citricola* zoospore behavior—taking into account the effects of surface tension, initial cell concentration, and suspension depth—suggests that the pattern swimming is an example of bioconvection, an overturning instability induced when the upper regions of a fluid become denser than the lower regions [17]. For zoospores, bioconvection involves (i) density instability because of the upward-swimming tendency of the zoospores and (ii) gyrotaxis, which is the directed motion resulting from the orientation of cells by balancing gravitational and viscous torques [18]. Another series of experiments combined with mathematical modelling were conducted to investigate the auto-aggregation behavior in *P*. *infestans* zoospores. This work supports the hypothesis of a combination of bioconvection and chemotaxis operating sequentially to achieve auto-aggregation. First, bioconvection causes the rapid formation of plumes. Then, chemotaxis between plumes is required to form larger auto-aggregates over a longer timescale [19]. A better understanding of the driving forces generated by bioconvection and of molecular mechanisms governing zoospore chemotaxis should help to define the conditions required for collective recruitment in the early phases of host infection.

## What Are the Known Modes for Amplification or Inhibition of Adhesion to the Host?

After the motile period and the attraction process, a transition from motile zoospore to nonmotile cyst occurs on the host cell surface. It is characterized by the loss of flagella, the elaboration of a primary cell wall, and the secretion of adhesive molecules [20]. The polyphagous species *Phytophthora parasitica* may use zoospore communication to amplify and increase local adhesion by means of biofilm formation. A cluster of founder cells adheres at the same site of infection and emits an unknown signal. Then, the oriented migration of additional waves of zoospores leads to biofilm formation by local and massive encystment. The structure is embedded within an extracellular mucilage and is speckled with channels in which swimming zoospores circulate [21]. The biofilm transcriptome is characterized by the coordinated up-regulation of a set of genes encoding mucin-like proteins, exporters and/or importers of substrates, and RXLR effectors [22,23] suggesting that the biofilm contributes to the dynamics of recruitment of effector functions to optimize infection effectiveness. Diverse lines of further research need exploration with respect to addressing structural and functional aspects of the biofilm, especially in natural habitats of oomycetes. For instance, how do zoospores deal with biosurfactants produced in the soil by bacteria such as *Pseudomonas* [24] to succeed in aggregation at the host surface? Does the biofilm increase the survival and resistance of oomycetes to environmental variations (temperature, hydric potential, and salinity) and/or to (bio)chemical stresses (microbial antibiosis activities, plant defense responses, or anthropogenic treatments against crop diseases)? Is it a structure favoring exchanges of signals and/or nutrients between sessile, biofilm-associated cells and planktonic zoospores or other circulating microorganisms? Is it involved in mediating competition or cooperation occurring at the surface of host cells between oomycetes and other pathogens or opportunistic microorganisms?

The first investigations of the metainteraction between an oomycete and a resident microbiota have explored fish microbiota and *Saprolegnia*-associated egg disease [25,26]. These studies show that attachment of *Saprolegnia* species on fish eggs is regulated by interkingdom interactions between the oomycete and bacterial microbiota. A metataxonomic analysis indicated a correlation between a low incidence of saprolegniosis on salmon eggs having an immature adaptive immune system and a high richness and abundance of specific commensal Actinobacteria. Bacteria from the genus *Frondihabitans* effectively inhibit attachment of *Saprolegnia* to eggs [25]. Another study established that within Gammaproteobacteria, Pseudomonadaceae represents one of the largest bacterial families associated with salmon eggs from a hatchery. *Pseudomonas* isolates from the microbiota associated with salmon eggs reduced egg mortality caused by *Saprolegnia diclina* [26]. Thus, the prokaryotic microbiota appears as one of the determining factors in establishing infection.

## How Do Members of a Resident Microbiota Regulate Cyst Germination and Hyphal Elongation?

Following encystment, a germination tube emerges that becomes firmly attached to the host surface. Different case studies report that microorganisms growing in the rhizosphere may exhibit antigerminative properties against plant pathogens. The oomycete *Pythium oligandrum*, licensed as a biocontrol agent, is a parasite of *Phytophthora* and *Pythium* species. The mechanism of inhibition consists of a nutrient and/or space competition toward *Pythium ultimum* in the cotton or sugar beet rhizosphere [27]. It involves the production of hydrolytic enzymes (e.g., cellulases) and the deposition onto the inner cell surface of *Phytophthora parasitica*, the causal agent of the black shank disease [28]. The bacteria *Enterobacter cloacae* suppresses *Pythium ultimum* seed rot by competition with the oomycete for plant-derived unsaturated long-chain fatty acids. A genetic approach pointed out the role of two bacterial genes in nutrient competition: *fadB*, encoding a subunit of β-oxidation enzymes, and *fadL*, encoding an outer membrane protein involved in the binding and transport of fatty acids into the cell. This competition leads to inhibition of oomycete germination and disease suppression [29].

## Do Microbial Partners Contribute to Penetration and Invasive Growth into the Host?

Pathogenicity of oomycetes also depends on their ability to enter into host tissues. A coinfection by two oomycetes can potentiate infection strategies among which there is gene expression for secreted effector proteins that manipulate structure, signaling, and metabolism of the host. *Albugo laibachii* infection enables colonization of the nonhost plant *Arabidopsis thaliana* by *Phytophthora infestans*. The set of *P*. *infestans* effector genes induced during the tripartite interaction overlaps with the genes induced in the host plant *Solanum tuberosum*. The penetration of *P*. *infestans* into *A*. *thaliana* tissues does not induce cell death associated with the hypersensitive response, a mechanism used by plants to prevent the spread of infection. The authors of this study also report observation of *A*. *laibachii* and *P*. *infestans* haustoria in the same plant cell [30]. Analysis of different multipartite interactions could help to understand how coinfection of host cells allows some oomycetes to act as opportunistic pathogens.

After successful penetration, the ability for invasive hyphal growth by elongation and ramification through the host tissue determines disease incidence. Different interspecific activities of extracellular products increase both infection efficiency and disease incidence rate. Supernatants conditioned by zoopsores of four species (*Phytophthora capsici*, *P*. *hydropathica*, *P*. *sojae*, and *P*. *nicotianae* [*P*. *parasitica*]) stimulate infection of each pathogenic species in three pathosystems (*Catharanthus roseus* cv. Little Bright Eye × *P*. *nicotianae*; *Lupinus polyphyllus* × *P*. *sojae*; *Glycine max* cv. Williams × *P*. *sojae*). The molecular basis of this cross signal remains to be determined [31]. Homoserine lactones such as AI-2 involved in interspecies communication between bacteria species [32] or involved in the quorum sensing in many bacterial species [33] could not be identified in the zoospore-conditioned supernatants [31]. Supernatants conditioned by the telluric bacterium *Bacillus megaterium* Sb5 stimulate both plant infection by the *Phytophthor*a species and up-regulation of effector gene expression in *P*. *sojae* [34].

## Is There a Contribution of Microbiota to Oomycete Propagule Dissemination in the Soil?

Rapid spreading of epidemics involves zoospore motility as the main dispersal mode for movement in soil. Other microbial species may contribute to a secondary mode of propagule dispersion through transitory physical association with pathogenic oomycetes. A motile unicellular *Vorticella* acts as a sower of propagules through a mutualistic interaction. The ciliate is able to colonize a *P*. *parasitica* biofilm, at which point it becomes sedentary, presumably to initiate a bacterial nutrition phase. When it again explores new habitats, it disseminates *P*. *parasitica* propagules of large size, which may include a sporangium. They are disseminated at high velocity, reaching up to 100 μm/s. They lead to the propagation of tobacco black shank disease, at least under laboratory conditions in a Boyden chamber system [35].

Until now, most of these studies have been performed under experimental conditions designed to mimic some of the more propitious aspects of natural habitats for the establishment of the host–oomycete interaction. An important challenge will be to now design sampling plans enabling the analysis of the functional capacity of microbiota and the evolutionary trajectories within microbiomes in natural habitats of oomycetes [36,37]. It is required to generate metagenomics and metatranscriptomics data in these conditions, in particular to explore the effector gene repertoire [38] in order to determine the contribution not only of the coevolution between *R* gene and effector pairs but also of the pleiotropic aspects of the microbiota–oomycete coevolution [13] to the maintenance of effector gene polymorphisms in oomycete populations [39]. On the other hand, a greater understanding of the microbial partnerships of oomycetes constitutes a vector for innovations in protection against diseases. The physics and biology of collective zoospore motion should help to elaborate biomimic materials for early monitoring of pathogenic populations in agrosystems [40, 41]. Phylogenetic, ecological, and functional characterization of the oomycete–microbiota network, in combination with analyses of shifts in plant microbiota composition in disease and healthy states, will contribute to get new insights in epidemiology. They should lead to the definition of biotic factors favorable to environmental distribution of inocula and to disease circulation. They will also be conducive to the development of microbiota-based strategies after setting the composition for new biocontrol products and the conditions of application [42, 43].
